# Progressive Ureteropelvic Junction Obstruction and Renal Function Deterioration in Adult, Even in a Short Period of Time

**DOI:** 10.1155/2023/6855975

**Published:** 2023-08-11

**Authors:** Abbas Basiri, Behzad Narouie, Saeed Reza Ghanbarizadeh, Hamidreza Rouientan, Mohadese Ahmadzade

**Affiliations:** ^1^Department of Urology, Urology and Nephrology Research Center, Shahid Labbafinejad Medical Center, Shahid Beheshti University of Medical Sciences, Tehran, Iran; ^2^Department of Urology, Zahedan University of Medical Sciences, Zahedan, Iran

## Abstract

**Introduction:**

The ureteropelvic junction obstruction is more common in children; however, it can also affect adults. The management of this condition has shifted toward a nonoperative approach with serial ultrasonography and renography. *Case Presentation*. The ureteropelvic junction obstruction imaging with significant renal function deterioration in an adult patient is described in this report. Laparoscopic exploration revealed aberrant vessels that compress the ureteropelvic junction against the lower pole of the kidney.

**Conclusion:**

It is important to consider that some of the ureteropelvic junction obstruction cases can get worse even in a short period of time.

## 1. Introduction

Hydronephrosis related to ureteropelvic junction obstruction (UPJO) is more commonly diagnosed in children but can also occur in adults [1]. A junctional obstruction is identified by imaging (computerized tomography- (CT-) urography), and a poor drainage pattern is detected by a diuretic renogram (DRG) [2]. Management of this condition focuses on relieving symptoms and preventing renal impairment. This disorder has undergone significant changes over the last decade, with the initial approach being a nonoperative one, accompanied by serial ultrasonography and renography to monitor the degree of hydronephrosis and renal function on a regular basis [1, 3]. Many cases of UPJO will improve over time without intervention. Conversely, in this report, we describe an adult case without definitive imaging finding of UPJO, but abruptly progressive imaging of UPJO with significant renal function deterioration.

## 2. Case Presentation

**Figure 1:**
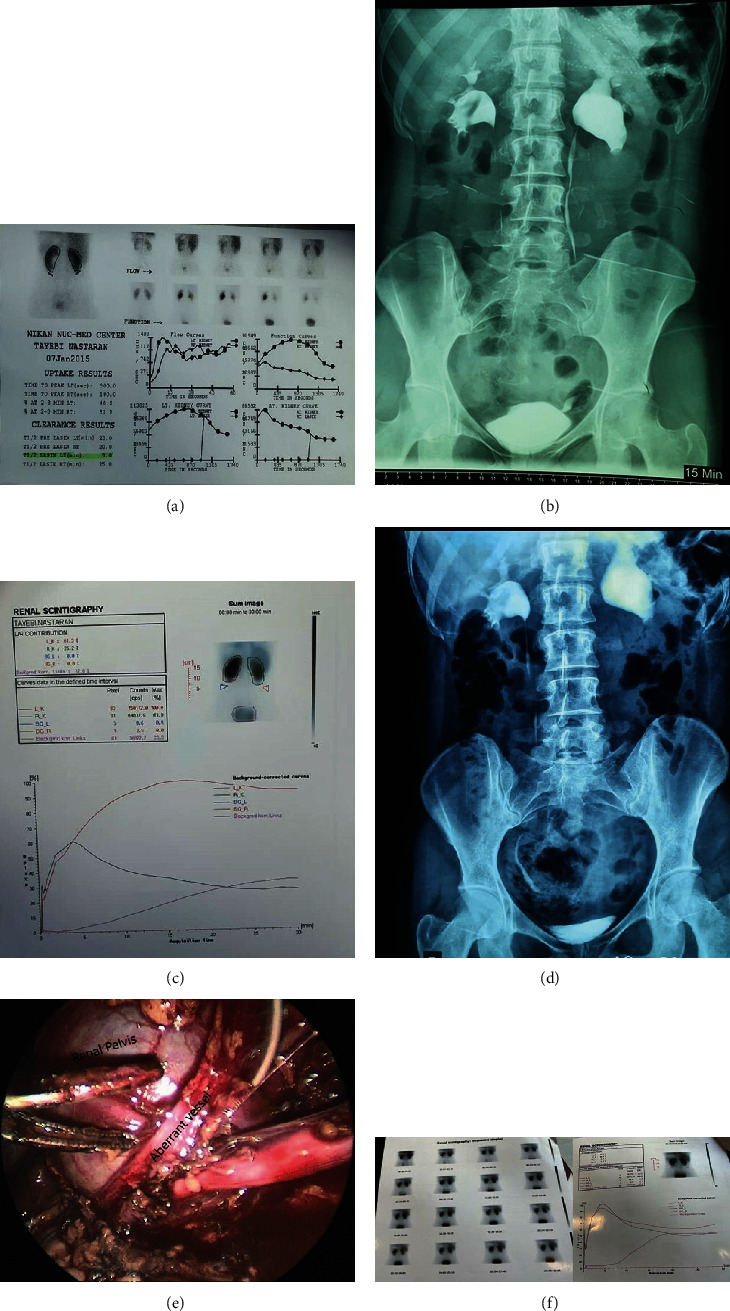
(a) Dilated left pyelocaliceal system with no significant obstruction. (b) Intravenous pyelogram showed no significant obstruction on the left side. (c) Hydronephrosis with obstruction pattern on the left side. (d) Intravenous pyelogram showed significant obstruction on the left side. (e) A laparoscopic image showing an aberrant vessel. (f) Normal functioning with evidences of partial obstruction in the left side.

A 32-year-old woman with a previous history of left mild hydronephrosis, nonspecific intermittent left flank pain, and equivocal DRG result was on close observation (Figures 1(a) and 1(b)).

After 7 years of surveillance and ruling out the vesicoureteral reflux, her symptoms got worsen during 6 months with remarkable changes in her imaging findings (Figures 1(c) and 1(d)). Throughout this period, the patient did not have any documented history of significant weight loss, drastic changes in BMI, or the occurrence of kidney stones.

Having been referred to our center, she was scheduled for transperitoneal laparoscopic evaluation due to persistent pain and obvious deterioration in renal function. There was an aberrant vessel during laparoscopy, and after releasing and transpositioning this vessel, the left renal pelvis showed an ever-increasing dilation, which meant an intrinsic obstruction at UPJ may be responsible for the situation (Figure 1(e)). So, we performed dismembered pyeloplasty with no early complication, and after three months, she noted great relief in her symptoms, as well as that the follow-up DRG demonstrated obvious improvement in renal function (Figure 1(f)).

## 3. Discussion

UPJO is increasingly being diagnosed prenatally through ultrasonographic screening. The condition can be due to intrinsic or extrinsic factors and can be congenital or acquired. As a result of UPJO, there are several causes, including ureteral kinking, an aperistaltic segment, or a high insertion of the ureter, respectively [4]. However, UPJO can also be caused by one or more of these causes as well [1]. In spite of prenatal screening, UPJO is still diagnosed in adulthood incidentally as renal colic, urinary lithiasis, hydronephrosis, and pyelonephritis. The presence of hydronephrosis is not necessarily indicative of ongoing high-grade obstruction, so further investigation is necessary to confirm the diagnosis. Surgery was the gold standard for treating it, but it is increasingly being managed conservatively in pediatric practice, with careful monitoring of pelvic dilation, renal function, and clinical symptoms [1]. Studies on the conservative treatment of UPJO in adults have shown a stable condition in the absence of complications; therefore, surgical treatment should only be considered for patients with recurrent flank pain or urinary infections [5]. Nevertheless, our report represents a somewhat unique situation in which patients had a worsening of both hydronephrosis and renal function in a short period of time over the course of surveillance. Despite this, the potential benefits of surgical treatment should still be carefully weighed against the potential risks before making a decision, as any unnecessary surgery should be avoided.

An active surveillance study conducted by Gulur et al. found that during follow-up, adults diagnosed with UPJO had significantly reduced renal function without developing any symptoms [6]. In the same series, however, none of the patients with equivocal UPJO showed signs of renal function deterioration after a median follow-up of 29 months.

There is a wide spectrum of UPJO, ranging from a reversible radiological finding, which occurs without producing renal damage, to well-defined histological alterations of the UPJ, which are associated with clinically significant renal parenchyma injury. It remains unclear whether the parenchymal damage in congenital UPJO is caused by chronic obstruction or genetically histological alterations in the renal parenchyma independent of obstruction degree [7]. In addition, the improvement of excretory capacity after a pyeloplasty is not always accompanied by a return of renal function [8]. In order to determine the best management and follow-up paradigm for UPJO, more studies are required on the natural history of hydronephrotic kidneys with poor drainage and decreased function.

## 4. Conclusions

There is a lack of consensus on the frequency of diuretic renography during follow-up, which should be into consideration to ensure appropriate and timely intervention in the event of renal function deterioration. While taking a conservative approach to asymptomatic UPJO in adult patients seems reasonable, some cases may experience worsening even in a short period of time. More studies are required to determine the best management and follow-up protocols for UPJO in adult patients.

## Data Availability

The data used to support the findings of this study are included within the article.

## Abbreviations

UPJO: Ureteropelvic junction obstruction
CT: Computed tomography
DRG: Diuretic renogram.
